# Demographic and Clinical Characteristics of Completed Suicides in Mexico City 2014–2015

**DOI:** 10.3389/fpsyt.2018.00402

**Published:** 2018-09-07

**Authors:** Ana L. Romero-Pimentel, Roberto C. Mendoza-Morales, Ana Fresan, Fernando Garcia-Dolores, Eli E. Gonzalez-Saenz, Mirna E. Morales-Marin, Humberto Nicolini, Guilherme Borges

**Affiliations:** ^1^Facultad de Psicología, Universidad Nacional Autónoma de Mexico, Ciudad de Mexico, Mexico; ^2^Instituto Nacional de Medicina Genómica, Ciudad de Mexico, Mexico; ^3^Instituto de Ciencias Forenses, Tribunal Superior de Justicia de la CDMX, Ciudad de Mexico, Mexico; ^4^Instituto Nacional de Psiquiatría Ramón de la Fuente Muñiz, Ciudad de Mexico, Mexico; ^5^Hospital Psiquiátrico Fray Bernardino Álvarez, Ciudad de Mexico, Mexico

**Keywords:** suicide, sex, epidemiology, coroners, Mexico City

## Abstract

**Objective:** To analyze sex differences in demographic and clinical characteristics of individuals who died by suicide in Mexico City.

**Method:** Statistical analysis of residents of Mexico City whose cause of death was suicide, during two years period from January 2014 to December 2015, with a coroner's report. Suicide mortality rates were calculated by age, sex, and location within the city. The Chi-squared test was used to assess statistical differences.

**Results:** From January 2014 to December 2015, 990 residents of Mexico City died by suicide (men: 78.28%, women: 21.72%). Among males, the highest mortality rates were among the groups of 20–24 and 75–79 years old, whereas in women, the group with the highest mortality rate was 15 to 19 years old. 74% of the sample used hanging as suicide method. However, men had higher rates of a positive result in the toxicology test (40%) (*p* < 0.05). There was no concordance between male and female suicide by city jurisdictions.

**Conclusion:** Our results provide evidence that the characteristics of Mexico City's residents who committed suicide had significant sex-related differences, including where they used to live. Understanding the contributory factors associated with completed suicide is essential for the development of effective preventive strategies.

## Introduction

Suicide is a major worldwide public concern that causes almost half of all violent deaths of males and 71% in the female population, which translates to about 800,000 suicides per year (1). In Mexico, suicide rates have been increasing over the past 40 years, affecting more men than women (2). The suicide rate for men increased from 5.95 to 8.50 (per 100,000 inhabitants) from 2000 to 2015, and from 1.06 to 2.00 for women (3, 4). Mexico City, one of the largest urban areas in the world, has one of the lowest suicide rates in the country: the mean standardized suicide rate was 4.1/100,000 for 2015 and 4.8/100,000 for 2014, being 6.8 for males and 1.7 for females in 2015 and 7.9 for males and 2.1 for females in 2014 (4, 5). Nevertheless, a rise of 14.30% has been observed in the number of suicides between 2000 and 2014 in Mexico City (4). In this context of increasing suicide rates, the study of suicide related to demographic and clinical risk factors has special relevance.

Several demographic risk factors associated with suicide have been clearly documented, for example, male sex, younger age, single, low income, and job loss (6–8). Actually, several studies have reported gender and age differences associated with suicide (6, 7, 9–14). In Mexico, very few studies focus on this area (3, 15, 16). A forensic study in the south of Mexico reported that more men die by suicide than woman (78 vs. 22%). Women decedents were older on the average than men. Also, women had more years of schooling, but their main occupation was housewife (37.5%). Men were most likely to be retired and were more likely to consume alcohol at the time of suicide (52.1%) (17).

The official sources for suicide data in Mexico come from the National Population Council (CONAPO; Spanish acronym) the national vital statistics system of the National Institute of Statistics and Geography, and the General Department of Health Information (Dirección General de Información en Salud) (DGIS). These institutions sometimes have discrepancies in their suicide data, probably due to differences in the classification criteria for deaths, in registration, and in filing inconsistences (18). The statistics they provided are useful, but they are insufficient to produce a summary profile of suicide cases, because they do not report the demographic characteristics of the deceased or the circumstances surrounding the deaths. In many countries, assessment of coroner or medical records is frequently used to gain insights on basic demographic data for suicide (14, 19–21). In Mexico, and in accordance with *National Code of Criminal Procedures* (22), a coroner must identify the causes of all uncertain or violent deaths, including all potential cases of suicide. Therefore, each possible suicide is subject to an investigation conducted by forensic pathologists and police officers, providing data to enable the coroner's office to generate a formal verdict regarding manner of death. The records from the coroner involving suicides generally include demographic information, circumstances of the death (as reported by witness reports), acute, and chronic stressful life situations, autopsy and toxicology reports, police investigation records, medical and psychiatric reports from hospitals, suicide notes, and insurance data. In Mexico City, the Forensic Science Institute (INCIFO) is the institution that garners all suicide cases for the inhabitants of the city. To the best of our knowledge, this information has not been used before to produce a profile of suicide victims in Mexico City.

Coroner's records provide an accessible source of information on suicides. Given that suicide is an increasing problem in Mexico, we believe that a better understanding of the demographic and clinical profiles of suicide victims can not only advance the research literature, but also could have important implications for suicide prevention, with a gender perspective. For these reasons, the aim of this study is to analyze the demographic and clinical characteristics such as history of suicide, previous history of suicide attempts, individual's use of drugs, and the presence or absence of a medical health problem of individuals who died by suicide in Mexico City, focusing on possible sex differences.

## Materials and methods

### Sample

We included all Mexico City resident who had a coroner's record for the cause of death, intentional self-harm (X60–X84) codes of the International Classification of Diseases, 10th Revision (ICD-10) during the 2 year period of January 2014 to December 2015. Mexico City is divided into 16 administrative jurisdictions, with a population of ~8,918,653 people (23). For confidentiality purposes, we assigned an unrepeatable and unidentifiable code to each coroner's records. None of the personal data of the sample were used in the decodification process. Furthermore, data were organized on a protected locked database stored at INCIFO, and only the principal investigators had access to it.

### Data sources

Data for our study were derived from the complete records prepared for each suicide case and kept in the Forensics Sciences Institute of Mexico City. The records from the coroner's office involving suicides included demographic information, circumstances of the death, acute and chronic stressful life situations, autopsy and toxicology reports, police investigation records, medical, and psychiatric reports from hospitals, suicide notes, insurance data, and death certification. Two researchers extracted information from these files, which must be consulted on site, and discussed possible sources of inconsistencies.

### Data collection questionnaire and variables

We developed a collecting data questionnaire based on suicide risk factors (24–26) that included demographic characteristics, clinical variables, and circumstances of death. Demographic variables included sex, age, years of education, marital status (married or single), occupational status, religion, and place of residence. Clinical variables contained the individual's use of legal and illegal drugs, his or her previous history of suicide attempts, and the presence or absence of a medical health problem. Medical health problem was succinctly presented by the attending forensic physician and later categorized into ICD-10 codes. Finally, circumstances of death were considered, such as death method, toxicology tests, place of occurrence (at home, public place, etc.), and whether a suicide note was found. This information, included in the coroner's records, was obtained through the victim's families and witness testimonies.

### Statistical analysis

Demographic, clinical, and suicide characteristics were reported based on frequencies and percentages for categorical variables and with means and standard deviations (SD) for continuous variables. For the comparison by sex, Chi-square (χ^2^) analyses for contingency tables were used for the comparison between men and women. Significance level for tests was established at *p* ≤ 0.05. Suicide mortality rates were calculated using the number of suicide deaths as the numerator and the 2014–2015 mid-period population by age, sex, and location for the study period as the denominator. Data of the mid-period population was obtained from the National Population Council, according to the population census from 2010, recorded by municipality, age, and sex (27). We analyzed our data by using the Graph Pad Prism, version 6.0 for Macintosh, statistical software package.

### Ethical considerations

According to the international guidelines of good clinical practices (GCP) and the Helsinki Declaration, anonymous databases can be used when confidentiality and absence of harm can be guaranteed (28). The Bio Ethical Committee for Human Research at Forensic Science, Institute of Mexico City, approved this secondary data analysis project.

## Results

### Suicide rate mortality by age and sex

**Figure 1:**
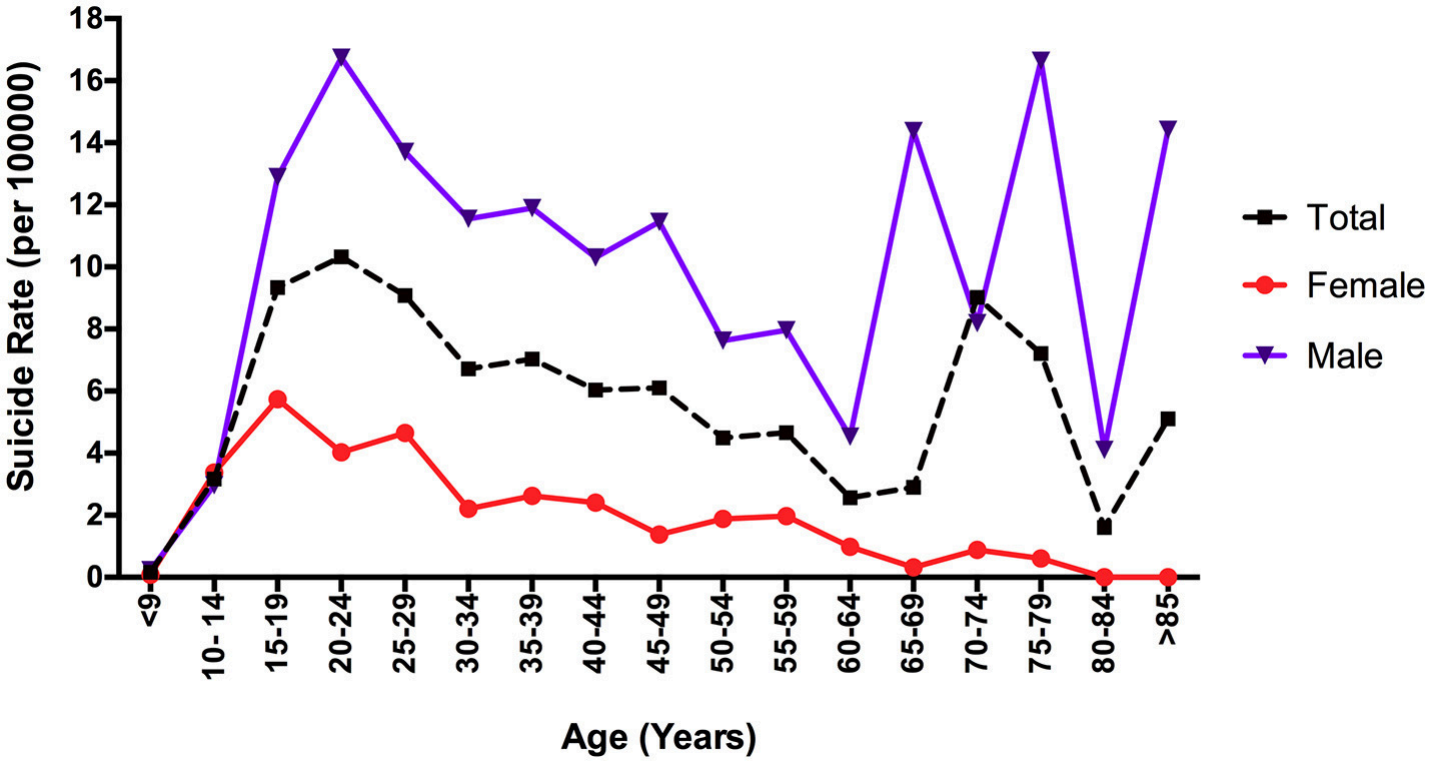
Suicide rate (per 100,000) in Mexico City by sex and age groups, 2014–2015.

Nine hundred and ninety deaths were identified as suicides between January 2014 and December 2015 in Mexico City: 775 men (78.28%) and 215 women (21.72%). Compared to women, men showed higher rates of suicide across all age groups, except for the 10–14-year-old group, in which female rates were higher. The highest suicide mortality rate for men included the following groups; 20–24 and 75–79 years (16.74/100,000 and 16.64/100,000, respectively). On the other hand, the women's group with the highest rate was the 15–19-year-old group (18.14/100,000) (Table 1, Figure 1). As is apparent from this figure, rates increased sharply for women 14–19 years old and then declined steadily. For men, on the other hand, the curve was bimodal, with high rates at the beginning of adulthood and among elderly men.

**Table 1:** Suicide rate (per 100,000) in Mexico City by sex and age groups, 2014–2015.

**Age (years)**	**Men**		**Suicide rate**	**Women**		**Suicide rate**	**Total**		**Suicide rate**
	***n***	**%**		***n***	**%**		***n***	**%**	
	**775**	**78.28**		**215**	**21.72**		**990**	**100**	
<9	3	0.39	0.23	1	0.47	0.08	4	0.40	0.16
10–14	20	2.58	2.97	22	10.23	3.38	42	4.24	3.17
15–19	89	11.48	12.89	39	18.14	5.74	128	12.93	9.34
20–24	118	15.23	16.74	29	13.49	4.03	147	14.85	10.32
25–29	96	12.39	13.71	34	15.81	4.65	130	13.13	9.08
30–34	78	10.06	11.55	16	7.44	2.21	94	9.49	6.72
35–39	78	10.06	11.90	19	8.84	2.62	97	9.80	7.03
40–44	64	8.26	10.3	17	7.91	2.41	80	8.18	6.03
45–49	64	8.26	11.45	9	4.19	1.38	74	7.37	6.10
50–54	37	4.77	7.63	11	5.12	1.88	48	4.85	4.49
55–59	33	4.26	7.96	10	4.65	1.97	43	4.34	4.66
60–64	15	1.94	4.53	4	1.86	0.98	19	1.92	2.57
65–69	35	4.52	14.38	1	0.47	0.32	16	1.62	2.90
70–74	14	1.81	8.19	2	0.93	0.88	36	3.64	9.02
75–79	19	2.45	16.64	1	0.47	0.61	20	2.02	7.21
80–84	3	0.39	4.11	0	0	0	3	0.30	1.61
>85	9	1.16	14.42	0	0	0	9	0.91	5.11

### Distribution of rates within Mexico City

**Figure 2:**
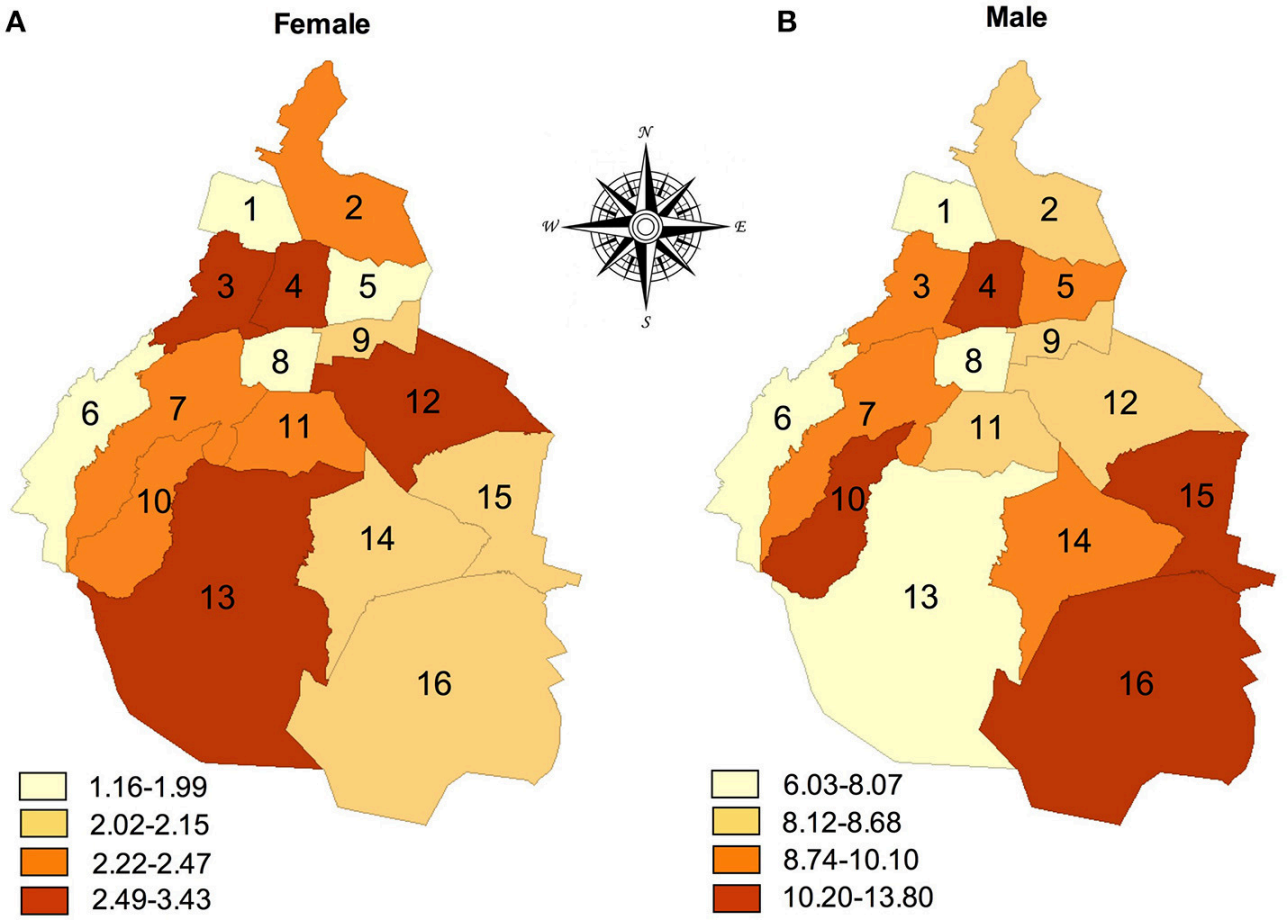
Suicide rates (per 100,000 population) in Mexico City, by sex and administrative jurisdictions, 2014–2015. The numbers indicate the administrative jurisdictions: 1 = Azcapotzalco, 2 = Gustavo A. Madero, 3 = Miguel Hidalgo, 4 = Cuauhtémoc, 5 = Venustiano Carranza, 6 = Cuajimalpa, 7 = Àlvaro Obregón, 8 = Benito Juárez, 9 = Iztacalco, 10 = La Magdalena Contreras, 11 = Coyoacán, 12 = Iztapalapa, 13 = Tlalpan, 14 = Xochimilco, 15 = Tláhuac, 16 = Milpa Alta.

The administrative jurisdictions with the highest suicide rate in Mexico City for men were: Cuauhtémoc (#4 = 13.82 per 100,000), La Magdalena Contreras (#10 = 11.67 per 100,000), Milpa Alta (#16 = 10.47 per 100,000), and Tláhuac (#15 = 10.15 per 100,000). For women, the peak suicide rate occurred in Miguel Hidalgo (#3 = 3.43 per 100,000), followed by Tlalpan (#13 = 3.16 per 100,000), Iztapalapa (#12 = 2.52 per 100,000), and Cuauhtémoc (#4 = 2.49 100,000) (Figure 2). In comparing both maps, only the jurisdiction of Cuauhtémoc had the highest rates of suicide for both sexes. We performed a simple correlation between administrative jurisdictions and suicide rates. We found that the distribution of deaths rates within Mexico City (16 Administrative Jurisdictions) were not similar for males and females (Spearman, Rho = 0.125, *n* = 16, *p* = 0.323). This means that the local distribution of suicide deaths by jurisdictions among males and females are different.

### Suicidal act characteristics

In Mexico City, the principal method of suicide for men and women was hanging (74.34%). There were differences for the second suicide method between groups; men used shooting with a firearm (13.42%), whereas women used jumping from a height (10.23%). Furthermore, a similar percentage between groups was found in reporting home as the most frequent place of occurrence (77.40% for men and 86% for women), followed by public places (13.50% for men and 8.40% for women). Woman suicides were much more likely to have had a prior suicide attempt than men. In our population, no differences were observed when we analyzed whether subjects left a written message (Table 2).

**Table 2:** Suicide characteristics by sex, 2014–2015.

	**Men**		**Women**		**Total**		**Statistics**
	***n* 775**	**% 78.28**	***n* 215**	**% 21.72**	***n* 990**	**% 100**	
**SUICIDE METHODS**							
Hanging	576	74.45	159	73.95	735	74.34	
Shooting with a firearm	104	13.42	10	4.65	114	11.52	
Poisoning	15	1.94	15	6.98	30	3.03	*x*^2^ = 30.26, *df* = 4, ***p*** < **0.0001**
Jumping from a height	45	5.81	22	10.23	67	6.77	
Others	35	4.52	9	4.19	44	4.34	
**PLACE OF OCCURRENCE**							
Home	601	77.55	185	86.05	786	79.30	
Public places	105	13.55	18	8.40	123	12.40	*x*^2^ = 7.43, *df* = 3, *p* = 0.059
Prison	17	2.19	3	1.40	20	2.00	
Other	52	6.71	9	4.20	61	6.30	
**PREVIOUS EPISODES OF SUICIDE ATTEMPT**							
No	676	87.20	164	76.30	840	84.85	
Yes	86	11.10	48	22.30	134	13.53	*x*^2^ = 18.13, *df* = 2, ***p*** < **0.0001**
Unknown	13	1.70	3	1.40	16	1.62	
**WRITTEN MESSAGE**							
No	653	84.40	168	77.70	821	82.93	
Yes	115	14.70	45	21.40	160	16.16	*x* ^2^ = 4.62, *df* = 2, *p* = 0.099
Unknown	7	0.90	2	0.90	9	0.91	

*Bold Values are significant at p ≤ 0.05*.

### Demographic and clinical characteristics

The amount of missing information in the clinical charts varied largely across variables, from 4.34% for a toxicological test to as much as 44.19% for marital status. With these caveats in mind, men's and women's suicides were different in some important variables. As shown in Table 3, men were more frequently employed at the time of death (65 vs. 19.3%) than women were. There were no differences in levels of education (9 years of education for both groups), marital status, religion, and medical health condition by sex. A further analyses of causes of medical conditions showed that the most common group of diseases was related to endocrine, nutritional, and metabolic systems (E00-E90) 29.10% in men and 23.91% in women, followed by diseases of the circulatory system 14.93% in men and 17.39% in women (data not shown). A marked use of alcohol was observed in the men group (54.3%) compared to the women group (24.7%). Nevertheless, there were more cases of smokers in the group of women (74.4%) than in the men group (55%). Last, regarding the toxicology test, more men than women turned out to be positive for substance use (40 vs. 18.14%, respectively). Among those positive for any substance, the principal substance used at time of death for men and woman was alcohol (83.09 and 74.36%, respectively), followed by cocaine (12.58%) and inhalants for men (8.06%) and sedatives (15.38%) and cocaine (12.82%) for women (data not shown).

**Table 3:** Demographic and clinical characteristics of suicide by sex, 2014–2015.

	**Men**		**Women**		**Total**		**Statistic**
	***n***	**%**	***n***	**%**	***n***	**%**	
	**775**	**78.28**	**215**	**21.72**	**990**	**100**	
**MARITAL STATUS**							
Married	196	25.30	39	18.10	235	18.14	
Single	258	33.30	81	37.70	339	37.67	*x*^2^ = 4.87, *df* = 2, *p* = 0.087
Unknown	321	41.40	95	44.20	416	44.19	
**OCCUPATIONAL STATUS**							
Housewife	5	0.60	57	26.50	62	6.26	
Student	82	10.50	54	25.60	136	13.74	
Employed	503	65.00	64	29.30	567	57.27	*x*^2^ = 250.07, *df* = 5, ***p*** < **0.0001**
Retired	33	4.30	4	1.90	37	3.74	
Unemployed	99	12.80	16	7.40	115	11.62	
Unknown	53	6.80	20	9.30	73	7.37	
**RELIGIOUS**							
Religious	569	73.50	153	70.70	722	72.80	
Non-religious	125	16.00	39	18.60	164	16.70	*x*^2^ = 0.53, *df* = 2, *p* = 0.76
Unknown	81	10.50	23	10.70	104	10.50	
**YEARS OF EDUCATION**							
6 years	171	21.90	34	15.80	205	20.60	
9 years	262	33.90	73	33.50	335	33.80	*x*^2^ = 7.9, *df* = 4, *p* = 0.09
12 years	194	25.00	67	31.20	261	26.40	
>12 years	100	12.90	33	15.80	134	13.50	
Unknown	47	6.30	8	3.70	55	5.70	
**SMOKERS**							
Yes	427	55.00	160	74.40	586	32.73	
No	284	36.70	39	18.10	324	59.19	*x*^2^ = 28.4, *df* = 2, ***p*** < **0.0001**
Unknown	64	8.30	16	7.50	80	8.08	
**ALCOHOL USERS**							
Yes	420	54.30	54	24.70	474	47.87	
No	302	38.80	149	69.70	451	45.56	*x*^2^ = 64.12, *df* = 2, ***p*** < **0.0001**
Unknown	53	6.90	12	5.60	65	6.57	
**MEDICAL CONDITION**							
Yes	134	17.30	46	21.40	180	18.20	*x*^2^ = 1.9, *df* = 1, *p* = 0.38
No	641	82.70	169	78.60	810	81.80	
**TOXICOLOGY TEST**							
Positive	310	40.00	39	18.14	349	35.25	
Negative	438	56.52	160	74.42	598	60.40	*x*^2^ = 37.82, *df* = 2, ***p*** < **0.0001**
Unknown	27	3.48	16	7.44	43	4.34	

*Bold Values are significant at p ≤ 0.05*.

## Discussion

The results of this study provide some of the first published data characterizing suicides in Mexico City, one of the most populated cities in the world, using forensics reports from coroner's office. The findings describe the demographic and clinical characteristics such as history of suicide, previous history of suicide attempts, individual's use of drugs, and medical health status of individuals. Brief discussions of some of the key areas follow.

In Mexico City, 990 deaths were attributed to and officially recorded as suicide in the years between 2014 and 2015. Men were 3.5 times as likely to commit suicide as women. This result is in accordance with previous studies about suicide in Mexico (2, 3, 17) and elsewhere (1). The suicide rate for men was high at the beginning of adulthood and among the elderly. On the other hand, suicide rates increased sharply for women and then declined steadily. These results have been well documented in other parts of the world, whereas the risk and patterns of suicide among young people and among the elderly are higher (29–34). It is important to mention that this finding agrees with the Mexican national suicide rate age trend (5). Unfortunately other regionals studies developed in Mexico did not pay attention to patterns of suicide among age groups, so we are not able to identify differences between Mexico City and other states (3, 15–17). There were no similarities between suicide deaths by sex and administrative jurisdictions within the city. It is not clear why some locations within the same city have different rankings for suicide rates for men than for women. It could be speculated that socioeconomic factors, lifestyles, access to education, and limited health care access might be implicated (35). Also, access to services, which increases the possibility of diagnosis and treatment of mental disorders, could have an impact on local suicide rates. Why these differences would affect more on sex is speculative at this moment. Further research is needed to explain these differences in the suicide rate in Mexico City.

The leading suicide method varies considerably among different societies. Completed suicides via firearm and hanging were ranked as the principal among residents of United States of America (36). Self-immolation is a common method for suicide in India, Pakistan and Sri Lanka (37). In Mexico, the most frequent suicide method used by men and women was hanging; as a second method, men used shooting by a firearm, whereas women used jumping from a height. This finding agrees with similar national Mexican studies (3, 15–17, 38). The disparity in suicide methods among countries may due to social, cultural and accessibility to certain methods used in suicidal acts. Based in Mexicans policies, the access to a firearm is restricted. Nevertheless, hanging and jumping from a height are easily accessible methods for Mexican population (10). These methods also have been considered violent and represent an indication of a strong determination to die (39). Home was the most frequent place of occurrence to die for both groups. It has been suggested that this is the place where suicide could be committed more easily, perhaps due to accessibility, planning, time, and privacy (17).

Comparatively, women were more likely to have had a prior suicide attempt than men (13 vs. 11.10%). This was expected because it has been reported before for both suicidal attempts and fatal suicide acts (9, 40). However, in our study, the proportion of people who have attempted suicide is lower than the one reported worldwide, in which approximately one third of suicides followed a prior attempt (41, 42). We did not find differences between men and women who left a written message. These findings are similar to Chávez-Hernández et al. (43). It has been reported that there are no significant demographic differences between those who leave notes and those who do not (44). Previous studies have shown that the presence of a suicide note is not associated with the level of suicidal intention (45). It is well known that the incidence of a suicide note varies considerably worldwide, ranging from 3 to 42% (46, 47). For instance, Kuwabara et al. (46) reported that in Japan, those who wrote notes were more likely to be women. Similarly, studies in Australia reported differences on the theme of the note, depending on the age (e.g., young women were more likely to write about themes related to escape from pain and romantic problems, whereas older women were more likely to write about anger toward others) (48). Unfortunately, we did not measure the themes of notes.

Marriage is generally thought to be a protective factor against suicide and gender plays a prominent role here (49). However, this factor seems to be associated different in Mexican suicides, to be married is the most prevalent status in suicides cases of Tabasco State population (17) whereas relatively more suicides were observed among singles in Mexico City, similar to studies reported before for Mexican general population (3) and elsewhere (12, 50–56). Increased suicide among singles has been attributed to lack of family ties and increased individualism (57). The association between the risk of suicide and years of schooling has been investigated, and the results indicate that there is a negative correlation between suicide and higher educational attainment (58–63). We found that the distribution of schooling in suicidal victims was lower than the general Mexico City population (9 vs. 15 years of schooling) (24). It has been documented that in low- and medium-income countries like Mexico, lower education is associated with risk of suicide (64, 65). Therefore, we suggest that in Mexico City, educational achievement is an important risk factor for suicide mortality in both men and women.

About 11% of the victims were unemployed. The association between unemployment and suicide is the most constant finding reported in previous studies (66). It has been suggested that to be unemployed could significantly increase economic problems, loss of working status, isolation, and low esteem; these could exert an influence to suicide (67, 68). Religion has been considerate as a protective factor against suicide (69). It has been suggested that spiritual beliefs may help people to develop better stresses cope strategies and provide sources of hope and meaning in life (70). Nevertheless, we found that religion is unrelated to suicide in Mexico City. The literature indicates that the relationship between religion and suicide risk is complex (71). Different religious affiliations provide different degrees of protection. We were not able to identify the type of affiliations or practices in our sample, limiting our interpretation.

We found a marked use of alcohol in men compared to women. Nearly 54% of male victims were alcohol users, and 40% of male victims turned out to be positive in the toxicology test at the time of death. It is well accepted that both chronic and acute substance use are associated with suicidal behavior (11, 72–74). Loss of inhibition, poor judgment, and impulsivity associated with excessive drinking may have triggered suicidal behavior (75, 76). Also, results from a Mexican National Comorbidity study suggest that substance use and impulse-control disorders are actually the strongest predictor in Mexico (77). Restricting the availability of certain drugs has proven an effective preventive measure of suicide (1).

**Figure 3:**
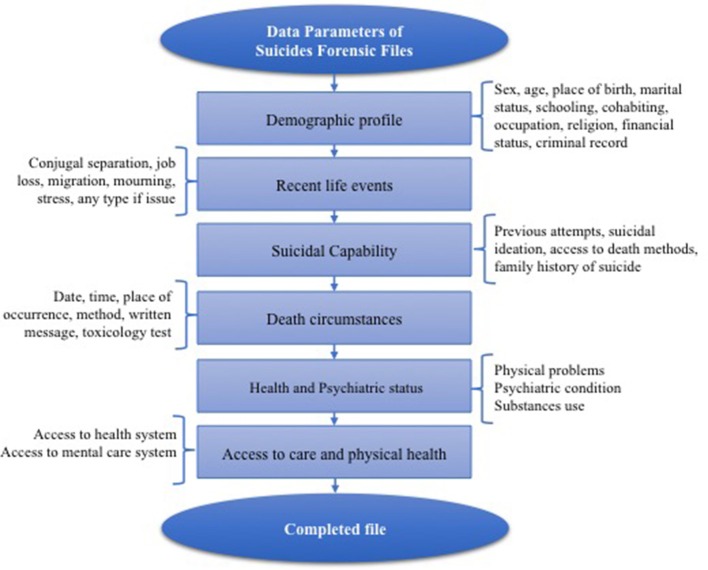
Data Parameters of Suicides Forensic Files.

The main factor that limited our conclusions was the high rate of missing data from the coroner's files. Unfortunately, coroner's records are often incomplete due to an absence of systematic and standardized procedures that they use to collect information in suicide cases. We suggest that the implementation of a standardized procedures data collection form that covers all parameters relevant to preventing suicide would help to reduce the amount of missing data in coroner's files (Figure 3). The full data collected by coroners in their investigations can be used at the local level to monitor suicide trends, may lead to develop a better-targeted suicidal prevention campaigns and help to identify individuals at high risk of suicide.

Furthermore, we were not able to compare our results with a matched group of controls. Finally, our results are representative of individuals in only one city of Mexico and should not be considered representative of other coroner's offices in other areas of the country. Selective suicide-prevention strategies that target specific populations by taking account local factors are a next step for researchers in Mexico.

## Conclusions

Our results represent an important step in identifying specific demographic and clinical circumstances surrounding suicide deaths in the largest city in Mexico. This information may help authorities develop specific prevention strategies based on sex differences and guidelines for those involved in the medical study of suicide.

## Author contributions

AR-P and GB study concept and design, data analysis and interpretation, drafting of manuscript, and drafting figures. AR-P, RM-M, and EG-S data collection and contributed to the study design. FG-D institutional support. AF, HN, and MM-M critical revision of article.

### Conflict of interest statement

The authors declare that the research was conducted in the absence of any commercial or financial relationships that could be construed as a potential conflict of interest.
